# Dr. William J. McDermott

**Published:** 1904-04

**Authors:** 


Dr. William J. McDermott, of New York, died March 12, 1904, aged 73 years. After graduation in 1854, at New York University, he settled in Westchester county, and was elected to the assembly in 1860. He resigned his seat to accept a commission as surgeon of the 66th N. Y. Infantry,—a regiment in which he was twice appointed surgeon. He resumed his practice at Westchester after the close of the war and so continued until his last illness.

